# Root traits confer grain yield advantages under terminal drought in chickpea (*Cicer arietinum* L.)

**DOI:** 10.1016/j.fcr.2016.11.004

**Published:** 2017-02-01

**Authors:** Purushothaman Ramamoorthy, Krishnamurthy Lakshmanan, Hari D. Upadhyaya, Vincent Vadez, Rajeev K. Varshney

**Affiliations:** a International Crops Research Institute for the Semi-Arid Tropics (ICRISAT), Patancheru, India; b Jawaharlal Nehru Technological University Hyderabad (JNTUH), Hyderabad, India; c Department of Agronomy, Kansas State University, Manhattan, KS 66506, United States; d UWA Institute of Agriculture, University of Western Australia, Crawley, WA 6009, Australia; e School of Plant Biology and Institute of Agriculture, The University of Western Australia, WA, Australia

**Keywords:** Deep roots, Drought tolerance, Field phenotyping, Root length density, Root:shoot ratio, Soil coring

## Abstract

Chickpea, the second most important legume crop, suffers major yield losses by terminal drought stress (DS). Stronger root system is known to enhance drought yields but this understanding remains controversial. To understand precisely the root traits contribution towards yield, 12 chickpea genotypes with well-known drought response were field evaluated under drought and optimal irrigation. Root traits, such as root length density (RLD), total root dry weight (RDW), deep root dry weight (deep RDW) and root:shoot ratio (RSR), were measured periodically by soil coring up to 1.2 m soil depth across drought treatments. Large variations were observed for RLD, RDW, deep RDW and RSR in both the drought treatments. DS increased RLD below 30 cm soil depth, deep RDW, RSR but decreased the root diameter. DS increased the genetic variation in RDW more at the penultimate soil depths. Genetic variation under drought was the widest for RLD ∼50 DAS, for deep RDW ∼50–75 DAS and for RSR at 35 DAS. Genotypes ICC 4958, ICC 8261, Annigeri, ICC 14799, ICC 283 and ICC 867 at vegetative stage and genotypes ICC 14778, ICCV 10, ICC 3325, ICC 14799 and ICC 1882 at the reproductive phase produced greater RLD. Path- and correlation coefficients revealed strong positive contributions of RLD after 45 DAS, deep RDW at vicinity of maturity and RSR at early podfill stages to yield under drought. Breeding for the best combination of profuse RLD at surface soil depths, and RDW at deeper soil layers, was proposed to be the best selection strategy, for an efficient water use and an enhanced terminal drought tolerance in chickpea.

## Highlights

• Root system development transcribed the drought yields of chickpea genotypes.
• In response to drought, roots turned more prolific, thinner & penetrated deeper soils.
• Highest drought tolerance depended on the strength of reproductive-phase root system.
• RLD after 45 DAS & deep RDW before maturity & early pod-fill RSR contributed to DS.
• Tolerance relied on maximum water acquisition enhancing shoot biomass & partitioning.

## 1 Introduction

Chickpea (*Cicer arietinum* L.) is the second most widely grown pulse globally, with a total production of 14.2 million tons from an area of 14.8 million ha and a productivity of 0.96 t ha^−1^ (FAOSTAT, 2014). The major chickpea producing countries include India, Australia, Pakistan, Turkey, Myanmar, Ethiopia, Iran, Mexico, Canada, and the United States. India, the largest chickpea producing country, accounts for about 68% of the global production. Its seeds are protein-rich alternatives of animal protein in human diet. Chickpea is a good source of protein (20–22%), and is rich in carbohydrates (around 60%), dietary fiber, minerals and vitamins (Williams and Singh, 1987, Jukanti et al., 2012). There is a growing international demand for chickpea and the number of chickpea importing countries has increased from about 60 in 1989 to over 140 in 2009. This is partly due to an increased awareness about the health benefits of pulses, such as influences on cardiovascular diseases, type 2 diabetes, digestive diseases, and some forms of cancer (Jukanti et al., 2012).

Chickpea is largely grown as a rain fed crop in the arid and semi-arid environments in Asia and Africa where more than 80% of the annual rainfall is received during the preceding rainy season (June–September). In these regions the rainfall variability is usually high, leading to varying amounts of water storage in the soil and varying intensities of drought stress (DS). Terminal drought is one of the major abiotic stresses limiting crop yield in chickpea. Chickpea is usually sown under stored soil moisture, with very little rainfall during the cropping season, leading to a constantly receding soil water condition. Such a growing condition imposes increasing intensities of water deficit as the crop cycle advances, leading to a severe water deficit at crop maturity. These types of receding soil water conditions impose a ceiling on the cropping duration demanding selection for matching duration varieties for the best adaptability and productivity (Saxena, 1987, Ludlow and Muchow, 1990).

Genetic improvement for better drought adaptation can be a long-lasting and less-expensive solution for drought management than the agronomic options. However, understanding yield maintenance under DS becomes increasingly difficult (Tuberosa and Salvi, 2006), due to the numerous mechanisms that plants can employ to maintain growth under low water supply. As a result, a trait-based breeding approach is being increasingly emphasized over yield-based breeding for realizing better stability as grain yields are heavily influenced by high genotype × environment (G × E) interactions and exhibit low heritability (h^2^) (Ludlow and Muchow, 1990). Also, a trait-based breeding increases the probability of crosses resulting in additive gene action (Reynolds and Trethowan, 2007, Wasson et al., 2012). Breeding for drought tolerance requires knowledge of the type and intensity of DS and the various traits and mechanisms employed by the plant to sustain productivity under terminal DS such as deep root system, increased partitioning coefficient and conservative water use without reducing the shoot biomass production.

The impact of various root traits on drought tolerance were found to be high under terminal DS environment, especially in environment where plant solely depend on the stored soil moisture (Ludlow and Muchow, 1990, Saxena et al., 1993, Krishnamurthy et al., 2003, Kashiwagi et al., 2006, Subbarao et al., 1995, Turner et al., 2001, Passioura, 2006, Wasson et al., 2014). For instance, Kirkegaard et al. (2007) demonstrated through field-based direct root and soil water measurements, that a 30 cm rooting depth increase in root system can capture an extra 10 mm of deep soil water at the grain development stage and result in an extra 0.5 t grains per hectare. Large root system with greater root prolificacy and rooting depth, was shown to influence not only transpiration through soil moisture utilization but also shoot biomass production, harvest index (HI) under terminal DS (Kashiwagi et al., 2006, Kashiwagi et al., 2013, Zaman-Allah et al., 2011, Purushothaman et al., 2016a). On the contrary, a deeper and more profuse roots alone had been considered not that important for higher grain yields (Vadez et al., 2008) or as a needless biomass partitioning (Passioura, 1983) or as an unnecessary energy loss due to its vigorous respiration compared to the shoot system (Vanderwerf et al., 1988, Krauss and Deacon, 1994). But chickpea root growth in the field under drought had been shown to be suboptimal (Krishnamurthy et al., 1996, Krishnamurthy et al., 1998, Ali et al., 2002, Kashiwagi et al., 2006) and the expensive root respiration had been demonstrated to be limited to a small section of the actively water acquiring soil layer. Therefore, settling these contradictions demand precise and detailed research evidence on the contributions of root traits to terminal drought tolerance for a rational use of these set of traits.

Plant breeders, who realize the importance of root system contribution, are generally hesitant to consider root traits for selection as these traits carry low heritability, variable in expression across soils and soil water environments and the field measurements are labor-intensive (Tuberosa et al., 2002, Malamy, 2005, Lynch, 2007, Gaur et al., 2008). Association studies of the whole plant root system with the grain yield production may reveal a positive (Kell, 2011, Bishopp and Lynch, 2015) or negative or neutral association (CIAT, 2007, CIAT, 2008, Zaman-Allah et al., 2011, Schoppach et al., 2013) as all the segments of the whole root system (from surface to deep layer roots) are not actively involved in soil water extraction (Ali et al., 2002, Carvalho et al., 2014) due to variable soil water availability across soil depths (Purushothaman et al., 2016a). Such interactions deter researchers from arriving at the right conclusion on the contribution of root traits to grain yield. For a proper understanding of the details and to arrive at the right conclusion, it is essential to measure the root distribution at various soil horizons across the entire growth period at least under both DS and optimally irrigated (OI) environments.

Such detailed assessments of root distribution in previous chickpea studies were largely undertaken using root boxes, small containers, lysimeters and most of the experiments were not carried out up to maturity to access grain yield. Thus, field based root assessment in chickpea remains very limited (Krishnamurthy et al., 1998, Serraj et al., 2004, Kashiwagi et al., 2006, Wasson et al., 2014). Along with root traits, shoot related traits and efficient soil moisture utilization can also be equally important in conferring drought tolerance. The association of various putative shoot traits and their priority ranking based on their level of contribution to grain yield under drought had already been confirmed using a related set of data from this study (Purushothaman et al., 2016b). Also the soil water uptake, development of drought stress across the whole growth period and the association of soil water uptake with the rooting density across soil horizon in relation to the genotypes and their drought tolerance have been already described (Purushothaman et al., 2016b). Therefore, to fill the knowledge gap, the objectives of this paper remained as 1) to assess the variation in root traits of chickpea genotypes with variable documented drought responses across crop growth stages and soil depths under both drought stressed and optimally irrigated field conditions and 2) to identify the key root traits by relating the root system variation with the yield components across soil depths and growth stages for enhancing drought tolerance.

## 2 Materials and methods

### 2.1 Plant material and crop management

Twelve chickpea genotypes viz., ICC 4958, ICC 8261, ICC 867, ICC 3325, ICC 14778, ICC 14799, ICC 1882, ICC 283, ICC 3776, ICC 7184, Annigeri, and ICCV 10 with close phenology but good contrasts for root development, drought response and canopy temperature depression were chosen for this study (Supplementary Table 1). These were field-evaluated on a Vertisol (fine montmorillonitic isohyperthermic typic pallustert) during the post-rainy seasons of 2009–2010 and 2010–2011, at ICRISAT, Patancheru (17° 30′ N; 78° 16′ E; altitude 549 m) in peninsular India. The water holding capacity of this field in lower limit: upper limit was 0.26:0.40 cm^3^ cm^−3^ for the 0–15 cm soil layer, and 0.30:0.47 cm^3^ cm^−3^ for the 105–120 cm soil layer. The available soil water up to 120 cm depth observed in this study was 216 mm in 2009–10 and 207 mm in 2010–11 (Purushothaman et al., 2016a). The bulk density was 1.35 g cm^−3^ for the 0–15 cm soil layer and 1.42 g cm^−3^ for the 105–120 cm soil layer (El-Swaify et al., 1985). The field used was solarized using a polythene mulch during the preceding summer primarily to fully protect the crop from wilt causing fungi *Fusarium oxysporum* f. sp, among other benefits and damages (Chauhan et al., 1988).

The fields were prepared in to broad bed and furrows with 1.2 m wide beds flanked by 0.3 m furrows. Surface application and incorporation of 18 kg N ha^−1^ and 20 kg P ha^−1^ as di-ammonium phosphate were carried out. The experiment was conducted in a randomized complete block design (RCBD) with three replications. Seeds were treated with 0.5% Benlate^®^ (E.I. DuPont India Ltd., Gurgaon, India) + Thiram^®^ (Sudhama Chemicals Pvt. Ltd. Gujarat, India) mixture in both 2009–10 and 2010–11 seasons. The seeds were hand-sown manually at a depth of 2–3 cm maintaining a row to row distance of 30 cm and a plant to plant distance of 10 cm with in rows with a row length of 4 m on 31 October 2009 and 20 November 2010. About 82 seeds were used for each 4 m row and at 10 days after sowing (DAS) the plants were thinned maintaining a plant-to-plant spacing of 10 cm. Immediately after sowing, a 20 mm irrigation through sprinklers was applied to ensure uniform seedling emergence. Subsequently, plants were grown under two soil water treatments; rainfed (to impose terminal DS) and optimal irrigation (irrigated once in 15–20 days on the basis of previous experience). The plots were kept weed free by hand weeding and intensive protection were taken against pod borer (*Helicoverpa armigera*).

### 2.2 Root sample extraction and processing

Steel soil core tubes (50 mm in diameter) were used to collect soil sample up to 120 cm in each plots. Such samplings were done at 35 (mid-vegetative stage), 50 (early reproductive stage) and 80 DAS (close to maturity under DS) in 2009–2010. These samplings in 2010–11 were at 35 (mid-vegetative stage), 45 (late vegetative stage), 55 (early reproductive stage), 65 (mid-reproductive stage), 75 (late reproductive stage) and 90 DAS (close to maturity). Each sample comprised of two or three cores and all these cores were pooled depth-wise to increase the sample size. The extracted soil core was separated in to sub-cores of 15 cm each having 8 sub-cores out of 120 cm. The soil sample containing roots were soaked in water overnight, soil was mixed with tap water to form a suspension, and the roots were recovered by passing the soil-water suspension through a 2 mm wire mesh sieve. Chickpea roots were then separated from the organic debris and weed roots manually by floating the sample material on water in trays. Recovered roots were suspended in a transparent tray with 2–3 mm film of water for easy dispersion of roots and scanned using a scanner. Total RL of each sample was measured using the image analysis system (WinRhizo, Regent Instruments INC., Quebec, Canada). The roots were kept for oven drying at 70 °C for 72 h (to constant weight). RDW (g m^−3^) was estimated for each depth or for total depth separately. As the root sampling was done at 15 cm soil increments, presentation of a precise rooting depth of each genotype was not possible. Therefore, the deep RDW was estimated to use as a proxy trait for rooting depth. A total of ultimate two soil depth (15 + 15 cm) root dry weights, which differed for each sampling time, was calculated as deep RDW. RLD as cm cm^−3^ of soil was estimated from the RL of the sub-core as root length (cm)/**v**olume of soil core (cm^3^). Root:shoot ratio was calculated using root and shoot dry weights.

### 2.3 Crop phenology

By regular observation, the date when 50% or more of the plants in a plot flowered was recorded as days to 50% flowering time of the plot and when 80% of the pods in a plot were dried was recorded as days to maturity for each plot.

### 2.4 Final harvest

After the physiological maturity, plant aerial parts (shoot − fallen pinnules) were harvested from an area of 3.6 m × 8 rows in each plot in both the year. Total shoot dry weights of the harvested sample were recorded after oven drying till constant weight at 45 °C in draught air driers and the total shoot dry weights were recorded. This shoot dry weight was adjusted for an estimated 20% loss of dry matter as pinnule fall (Saxena, 1984, Williams and Saxena, 1991). Grain weights were recorded after threshing. HI (%) was calculated as 100 × (grain yield/total shoot biomass at maturity). Plants from 1 m × 2 rows were used for the estimation of pod number and seed number per m^2^, seed number pod^−1^ and their weights. 100-seed weight was estimated from these seed weight and numbers.

### 2.5 Statistical analysis

The replication-wise data observed for all the phenotypic traits at different crop growth stages in 2009–2010 and 2010–2011 were subjected to statistical analysis using one way ANOVA. Significance of means was estimated through F value for each trait. The means derived from the ANOVA were used for correlations, regressions using GenStat software (12th edition) and path coefficient analysis using MINITAB^®^ Release 14.1 software. Variance components due to genotypes (σ^2^_g_) and error (σ^2^_e_) and their standard errors were determined. Here, the treatment (drought) was treated as a fixed effect and the genotype (G) × Treatment (T) interaction as random. The variance due to (G) (σ^2^_g_) and G × T interaction (σ^2^_gT_) and their standard errors were determined. Broad sense heritability (h^2^) was estimated as h^2^ = σ^2^_g_/(σ^2^_g_ + (σ^2^_e_/r)) where r was the number of replications.

## 3 Results

### 3.1 Weather and drought patterns

In both the years, the rain received prior to the cropping season was >900 mm, well distributed and more than enough to ensure complete charging of the soil profile. Cessation of seasonal rainfall occurred at 3rd October in 2009–10 and 15th November in 2010–11. In-season rains summed up to 44 mm during 9–19 DAS in 2009–10 and 12.6 mm during 19–22 DAS in 2010–11 which delayed the onset of drought slightly but the terminal drought stress did built up (data not shown). There was another rain (39 mm) at 75 DAS during 2009–10, but at this stage under drought stress the early or medium maturing accessions crossed the stage of responsiveness. Overall, the minimum temperatures were higher, particularly during the critical third and fourth week of December (flowering and early-podding period), and maximum temperatures were lower during 2009–10 (Supplementary Fig. S1). Relatively cooler minimum temperatures and maximum temperatures at vegetative period were observed in 2010–11. The cumulative evaporation and VPD was higher in 2009–10 compared to 2010–11 (Supplementary Fig. S1).

Largely, the pattern and the rate of soil moisture depletion remained the same in both the years but the soil moisture depletion was very rapid in 2010–11 season in the initial two weeks (Supplementary Fig. S2) as a result of high soil evaporation and a marginally high VPD (Supplementary Fig. S1). However, the rain that followed at 18–22 DAS minimized the soil moisture depletion. Also this year the soil moisture at harvest was slightly high. There was a large rain at 75 DAS in 2009–10 which raised the surface soil moisture to some extent, benefitted the late genotypes under DS and adversely affected all the genotypes under optimally irrigated treatment but this reverted the soil to the usual dry condition within two weeks.

### 3.2 The extent of variation in root traits

There were large range of variations among the tested genotypes for average root length density (RLD), total root dry weight (RDW), deep root dry weight (deep RDW) and root:shoot ratio (RSR) measured at different days after sowings in both drought treatments and years (Table 1, Table 2, Table 3). The traits RLD and RDW were found to have a significant correlation with each other in most of the sampling times, therefore, RLD alone was presented in this study. Based on the drought treatment means, DS increased the RLD and deep RDW at all the stages of root measurement compared to OI in both the years except for the initial sampling at 35 DAS in 2009–10, and 65 and 90 DAS for RLD and 90 DAS for deep RDW in 2010–11. Similarly, RSR was also found to be higher under DS than the OI plots with few exceptions. The genotype × drought treatment interaction was significant for RLD across different stages of crop growth in both years except RLD at 55 DAS in 2010–11 (Table 1).

A 42 mm rain that was received on 17 and 18 days before the root sampling (35 DAS) in 2009–10, delayed the progression of stress development under DS thus leading to less difference between these two drought treatments. Among the extent of variation (the difference between the highest and the lowest) in RLD means of genotypes measured at different DAS, it was the highest at 50 DAS both under DS (0.109 cm cm^−3^) and OI (0.178 cm cm^−3^) in 2009–10 (data not shown). In 2010-11, it was the highest at 55 DAS under DS (0.178 cm cm^−3^) and at 90 DAS under OI (0.172 cm cm^−3^). The extent of variation for deep RDW was the highest at 50 DAS under DS (22.78 g m^−3^) and at 35 DAS under OI (16.74 g m^−3^) in 2009–10. In 2010–11, it was the highest at 75 DAS under DS (48.75 g m^−3^) and at 90 DAS under OI (27.97 g m^−3^) (data not shown). For RSR, it was the highest at 35 DAS under both drought treatment and years. The variation among the genotypes was significantly different at p = < 0.001.

Up to 45 DAS as in 2010–11 (before flowering), genotypes ICC 4958, ICC 8261, Annigeri and ICC 14799 in 2009–10 and also ICC 283 and ICC 867 in 2010–11 produced greater RLD than the mean, in at least once out of all root measurements under DS (Table 1). During the reproductive growth duration after the average 50% flowering, genotypes ICC 14778 and ICCV 10 in 2009–10 and also ICC 3325, ICC 14799, ICC 283, ICC 8261 and ICC 1882 in 2010–11 produced greater RLD than the mean, in at least once out of all root measurements, under DS (Table 1). The genotypes ICC 7184 and ICC 3776 produced lower RLD than the mean at both vegetative and reproductive stages under DS in both the years. Under OI, genotypes ICC 4958, ICC 8261, ICC 283, ICCV 10 and ICC 867 at vegetative stage and, genotypes Annigeri, ICC 14778, ICC 14799 and ICC 3325 at reproductive stage produced greater RLD than the mean, in at least once out of all root measurements, in both the years (Table 1).Genotypes ICC 3776 and ICC 7184 produced lesser RLD than the mean across stages and years as under DS. With a few exceptions, based on the root growth all the 12 genotypes can be grouped into four categories by the significant deviation from the RLD mean under DS: i) good vegetative stage root growth (ICC 4958 and ICC 8261), ii) good reproductive stage root growth (ICC 867, ICC 3325, ICC 14778, ICC 283, ICCV 10 and ICC 1882), iii) above average root growth across all the stages (ICC 14799 and Annigeri) and iv) poor root growth (ICC 7184 and ICC 3776) across all stages. Irrelevant of the crop growth stage, all the drought tolerant genotypes had a good root growth compared to the drought sensitive genotypes ICC 7184 and ICC 3776.

**Fig. 1:**
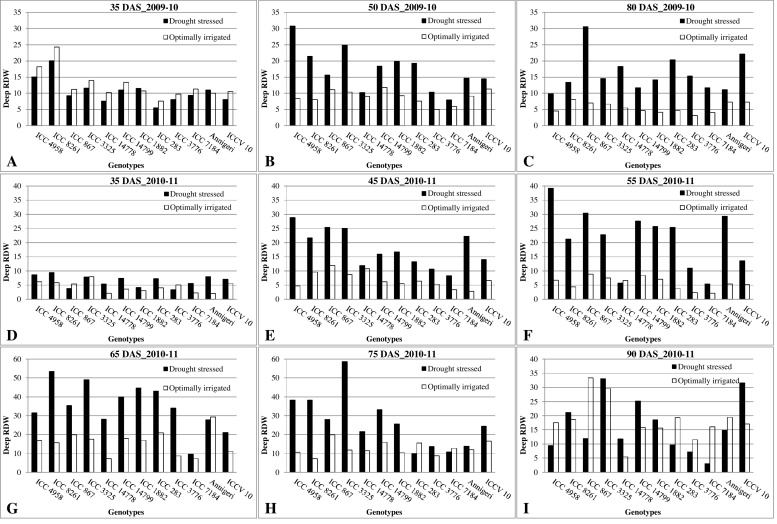
Deep root dry weight of 12 diverse genotypes of chickpea measured at different days after sowing (DAS) both under drought stress and optimal irrigation in a Vertisol during 2009–10 and 2010–11 post-rainy seasons.

Genotypes displayed significant differentiation among themselves for deep RDW across drought treatments and years (Fig. 1). In 2009–10, genotypes ICC 4958 and ICC 8261 had produced higher deep RDW at 35 DAS (Fig. 1A) and remained as higher deep RDW producing genotypes up to 50 DAS under drought stress (Fig. 1B). After 35 DAS, the drought tolerant genotype ICC 3325 was tend to have a relatively vigorous root penetration which resulted in higher deep RDW at 50 DAS (Fig. 1B). At the same time, the drought sensitive genotypes (ICC 3776 and ICC 7184), ICCV 10 and ICC 14778 were found to have below average deep RDW at 35 DAS and followed the similar pattern of deep RDW production up to 50 DAS except the best adapted genotype ICCV 10 (Fig. 1A and B). Under OI, the production of deep RDW was marginally high compared to DS at 35 DAS and this response was inexplicable as there were no imposed treatment differences at this sampling time (Fig. 1A). DS increased the deep RDW production about 1 to 2-folds higher compared to OI at 50 DAS (Fig. 1B) and this reduction was apparently in response to the irrigation provided at 38 DAS under OI.

Genotypes ICC 867, ICCV 10 and ICC 283 produced higher deep RDW at 80 DAS (Fig. 1C). Genotypes ICC 4958, ICC 14799, ICC 7184 and Annigeri had produced very low deep RDW. At this sampling stage, ICC 4958 and Annigeri were found to possess very low deep RDW as these were about to attain the physiological maturity leading to the death of roots. The highly drought tolerant genotype ICC 14778 which was found to be poor at the vegetative stage (Fig. 1A) became moderate in deep RDW production at this stage indicating the adaptive necessity. Under OI, deep RDW was reduced by about 1 to 4-fold compared to the DS and also the genotypic differentiation for deep RDW turned out to be very minor.

In 2010–11, all the genotypes produced high deep RDW at 35 DAS under DS compared to OI (Fig. 1D). Under OI the roots never descended below 45 cm in any of the genotype. Genotypes ICC 8261, ICC 4958, Annigeri and ICC 3325 had produced higher deep RDW at 35 DAS (Fig. 1D) and remained as higher deep RDW producing genotypes up to 45 DAS under drought stress (Fig. 1E). The genotypes ICC 1882, ICC 3776 and ICC 867 had produced very low deep RDW at 35 DAS while these were ICC 3776, ICC 7184, ICC 14778, ICC 283 and ICCV 10 at 45 DAS. Interestingly at this stage, the highly drought tolerant genotype ICC 867, which was very poor at 35 DAS (Fig. 1D), became one of the highest deep RDW producer and stood second in deep RDW production (Fig. 1E). At 45 DAS, DS increased deep RDW from one- to many-fold compared to OI. Also under OI, majority of the drought tolerant genotypes fell in the moderate to high deep RDW producing group. Both the drought sensitive genotypes have produced low deep RDW at this stage as under DS showing their constitutive nature of root behavior. Genotypes ICC 4958, Annigeri and ICC 867 had maintained the higher deep RDW and the genotype ICC 14799 started to produce higher deep RDW at 55 DAS (Fig. 1F). The genotypes ICC 7184, ICC 3776, ICC 14778 and ICCV 10 had produced low deep RDW under DS. The drought sensitive genotypes ICC 3776 and ICC 7184 had consistently produced lower deep RDW and the remaining genotypes had produced moderate to high and the genotype ICC 867 was the highest deep RDW producer under OI.

Genotypes that produced moderately deep RDW at 55 DAS became higher producers at 65 DAS (Fig. 1G). Conversely, the genotypes that produced higher deep RDW at 55 DAS became moderate at 65 DAS. Genotypes Annigeri, ICCV 10 and ICC 7184 produced very low deep RDW and the genotypes ICC 283 and ICC 1882 had produced higher deep RDW at this stage. There was not much changes occurred in deep RDW at 75 DAS compared to the previous root observations except that the genotypes ICC 4958 and ICC 14799 that were moderate till 65 DAS became higher in deep RDW and the genotypes ICC 1882 and ICC 283 that were higher in deep RDW at 65 DAS became moderate and low at 75 DAS, respectively (Fig. 1H). Interestingly, up to this crop stage all the genotypes at least in one sampling time had produced higher deep RDW except for the drought sensitive ones and the highly drought tolerant ICC 14778 genotype. Under OI, the drought sensitive genotypes were still in the low level of deep RDW as found under DS at 65 DAS (Fig. 1G) and the genotypes ICC 4958 and ICC 1882 were found to have low deep RDW at 75 DAS (Fig. 1H).

At 90 DAS under DS, genotypes ICC 3325, ICC 14799 and ICCV 10 had produced higher and, genotypes ICC 4958, ICC 3776, ICC 7184 and ICC 283 had produced very low deep RDW (Fig. 1I). The remaining genotypes were moderate including the highly drought tolerant genotype ICC 14778 that were very low in deep RDW in the previous root measurements. Under OI, genotypes ICC 867 and ICC 3325 produced higher and, genotypes ICC 3776 and ICC 14778 produced very low deep RDW. The rest of the genotypes were moderate in deep RDW.

During vegetative stage, genotypes ICC 14799, ICC 14778 and ICC 4958 in 2009–10 and ICC 14799, ICC 283, ICC 3325, ICC 1882 and ICC 867 in 2010–11 produced greater RSR than the mean, in at least once out of all root measurements, under DS and, genotypes ICC 14778, ICC 4958, ICC 867, ICC 7184 and ICC 283 in 2009–10 and ICCV 10, ICC 283 and ICC 867 in 2010–11 produced greater RSR than the mean, in at least once out of all root measurements, under OI (Table 3). During reproductive stage, genotypes ICCV 10, ICC 8261 and ICC 1882 in 2009–10 and ICC 14778 and ICC 283 in 2010–11 produced greater RSR than the mean, in at least once out of all root measurements, under DS and, genotypes Annigeri, ICC 8261, ICC 14799, ICC 3325 in 2009–10 and together with ICC 14778, ICCV 10 and ICC 7184 in 2010–11 produced greater RSR than the mean, in at least once out of all root measurements, under OI (Table 3). Genotype ICC 14799 produced consistently higher RSR than the trial mean across growth stages under drought treatment in both the years. Genotype ICC 3776 produced consistently lower RSR than the trial mean across growth stages, drought treatment and years.

In both the years, the heritability of these traits were high and ranged from 0.661 to 0.972 for RLD, 0.510 to 0.850 for deep RDW and 0.383 to 0.852 for RSR under DS, and from 0.759 to 0.941 for RLD, 0.387 to 0.926 for deep RDW and 0.316 to 0.841 for RSR under OI (Table 1, Table 2, Table 3).

### 3.3 The extent of variation in crop phenology, shoot biomass, grain yield and its components

The overall means for each drought treatment across years showed that DS reduced the days to 50% flowering and days to maturity relatively (Table 4). Overall, DS hastened flowering by 5 days in 2009–10 and by 7 days in 2010–11 and the less hastening in 2009–10 was due to the early stage rainfall and the delay in stress buildup. However, DS hastened maturity by 21 days in 2009–10 and by 13 days in 2010–11. Genotypes varied significantly in days to 50% flowering and days to maturity both in 2009–10 and 2010–11. Genotypes ICC 4958 and Annigeri were the earliest while ICC 283 and ICC 1882 were little longer than the early ones. The remaining genotypes were medium in duration. The genotype × drought treatment interaction was found to be significant for crop phenology in both the years (Table 4). The heritability values were high for the days to 50% flowering and for days to maturity under DS whereas it turned out to be less and moderate when irrigated. This was mostly due to a rain that was received immediately after the last irrigation reproductive disturbance due to excessive vegetative growth and lodging in some genotypes.

Under DS, both the shoot biomass and the grain yield produced at maturity were slightly higher during 2009–10. DS reduced the grain yield by 4 and 45% and the shoot biomass by 46 and 47% at maturity during 2009–10 and 2010–11 seasons, respectively (Table 4). The meager reduction in grain yield in 2009–10 was more due to a poor irrigation response in the irrigated treatment caused by a rainfall immediately following the last irrigation. Highly significant variations were found for the shoot biomass as well as grain yield among the genotypes, except for shoot biomass in 2009-10, and these variations were about 1.5-fold for the shoot biomass at maturity and 2-fold for grain yield among the accessions tested under DS. Under OI these variations were about 1.2–1.3 fold for the shoot biomass and grain yield. The genotype × irrigation interaction was found to be significant for grain in the year 2010–11 (Table 4).

Under DS, the genotypes that produced greater shoot biomass were the early strong rooting kabuli ICC 8261, the drought tolerant ICC 14778 and the drought sensitive ICC 3776. Additionally in 2010–11, two other drought tolerant genotypes ICC 867 and ICC 3325 and the well adapted genotype ICCV 10 produced greater shoot biomass (Table 4). Early and weak rooted ICC 283 and the best adapted Annigeri have produced the least shoot biomass across the years. The genotypes that produced consistently greater grain yield under DS were the two drought-tolerant genotypes ICC 867 and ICC 14778 and the best adapted genotype ICCV 10. Early large rooting ICC 4958, drought tolerant ICC 3325 and another best adapted genotype Annigeri yielded higher only in 2010–11. And the genotypes that produced consistently lesser grain yield under DS were the two drought-sensitive genotypes ICC 3776 and ICC 7184 along with the kabuli ICC 8261.

Heritability indices were high for the grain yield and moderate for shoot biomass under both drought treatments and year (Table 4). In general, the HI was relatively poor under OI. In 2009–10 a mean HI of 47.9 under DS was reduced to 26.6 under OI. Similarly in 2010–11, it was 45.5 under DS compared to 43.8 under OI, indicating that DS enhanced the HI compared to OI in both the years and the enhancement was much higher in 2009–10 primarily due to over watering OI. The genotypic distribution for HI followed similar pattern as that of the grain yield under both drought treatments and years. The variation among the genotypes for HI was significant at <0.001 level and the heritability were also high across drought treatment and years.

### 3.4 Root traits contribution to grain yield

RLD (cm cm^−3^) measured at various depths and at various growth stages were assessed for their contribution to grain yield through path analysis. The path coefficient estimated through path analysis is a standardized partial regression coefficient and measures the direct influence of one variable upon another and permits separation of correlation coefficient into components of direct and indirect effects. Direct and indirect effects of path coefficient have equal priority to determine the association between trait and drought tolerance. In few cases, the indirect effect had enough strength to provide a significant correlation between a trait and drought tolerance even when the direct effect of that trait did not show significant impact on the correlation. However, for brevity, the direct effect of traits on grain yield alone was presented. Also, the effects of variables that ranged between −0.05–0.05 were considered to be null and were not discussed.

**Fig. 2:**
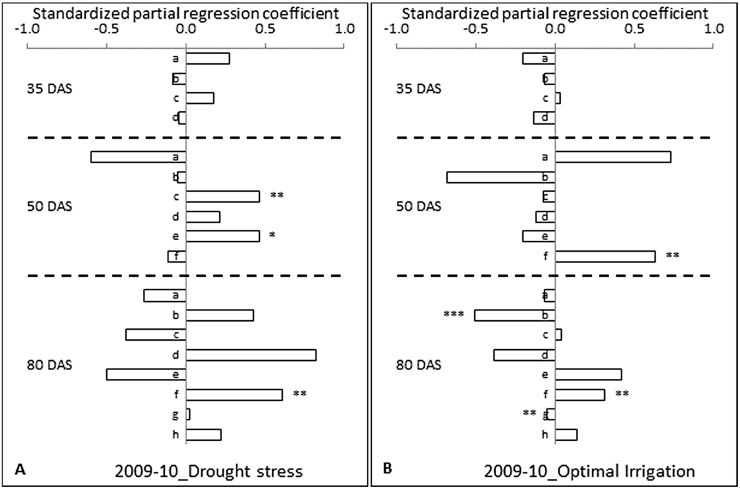
Standardized partial regression coefficients of root length density (RLD) measured at different days after sowing (DAS) on grain yield at maturity of 12 diverse genotypes of chickpea both under (A) drought stress and (B) optimal irrigation in a Vertisol during 2009–10 post-rainy season. The different number of asterisks on top of the bars denotes the different level of significant correlation between RLD and grain yield (*P < 0.05, **P < 0.01, ***P < 0.001). a = 0–15, b = 15–30, c = 30–45, d = 45–60, e = 60–75, f = 75–90, g = 90–105 and h = 105–120 cm soil depth.

Under DS in 2009–10, the RLD at 0–15 and 30–45 cm soil depths contributed to grain yield positively compared to the other two soil depths with minimum negative contribution at 35 DAS (Fig. 2A). Negative contributions had been largely an expression of reduction in RLD with advances in growth and in response to soil drying leading thus to a smaller RLD at the sampling time and a higher grain yield. At 50 DAS, the path coefficients revealed that RLD from all depths except 0–15 and 75–90 cm had provided positive contributions to grain yield. Particularly the RLD at 30–45 and 60–75 cm soil depths had a relatively larger direct contribution. Also RLD at these depths were positively correlated with the grain yield. At 80 DAS, the path coefficients of RLD from 15 to 30, 45–60, 75–90 and 105–120 cm exhibited a positive direct contribution to grain yield (Fig. 2A). The RLD of 45–60 cm soil depth had the highest direct contribution to grain yield and followed by 75–90, 15–30 and 105–120 cm soil depths. Among the RLD of different soil depths, the RLD at 75–90 cm soil depth had alone shown a significant positive association with the grain yield at p = < 0.01 level.

Under OI in 2009–10, the RLD in no soil depth had a positive direct effect on grain yield but the collective negative effect was large but not significant at 35 DAS (Fig. 2B). At 50 DAS, among the soil depths, RLD from 75 to 90 cm soil depth showed a significant positive correlation with the grain yield. At 80 DAS, the path coefficients of RLD from 30 to 45, 60–75, 75–90 and 105–120 cm had shown positive direct contribution to grain yield. The RLD of 60–75 cm soil depth had the highest direct positive contribution to grain yield followed by RLD of 75–90 (p = < 0.01) and 105–120 (p = < 0.05) cm soil depths. Though the direct contribution of roots from 90 to 105 cm is low, a positive significant correlation (p = < 0.01) was seen mainly through the indirect positive effects of adjacent soil depths. Across years and drought treatment, similar kind of indirect effect influence on direct effect resulting in positive correlation coefficient was also observed in few other sampling stages (data not shown).

In comparison to the OI under DS RLD at many depths, and mostly from the penultimate depths, provided positive contribution and RLD at these depths were also clearly associated with the grain yield through correlation. In contrast, under OI the direct effects were less in proportion and the RLD from ultimate rooting depths alone had contributed to yield.

**Fig. 3:**
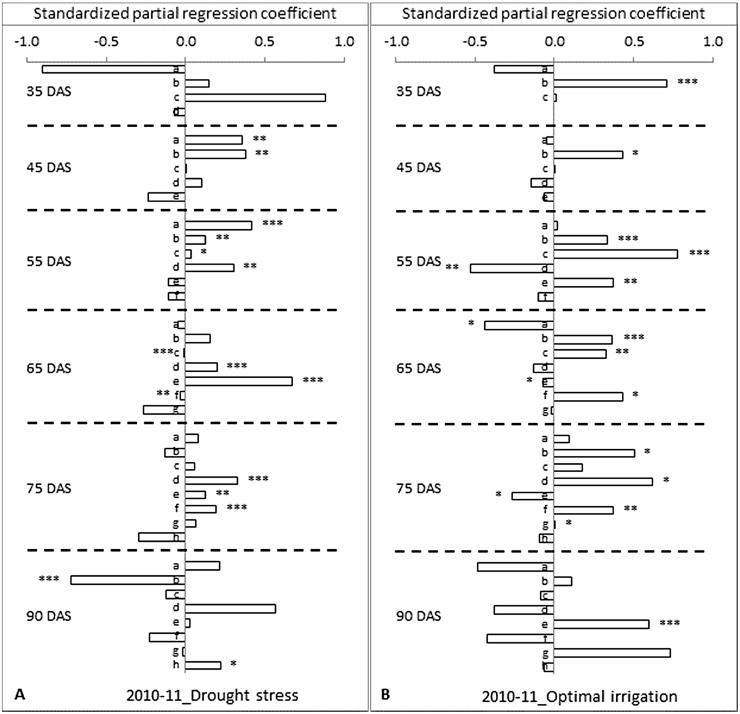
Standardized partial regression coefficients of root length density (RLD) measured at different days after sowing (DAS) on grain yield at maturity of 12 diverse genotypes of chickpea both under (A) drought stress and (B) optimal irrigation in a Vertisol during 2010–11 post-rainy season. The different number of asterisks on top of the bars denotes the different level of significant correlation between RLD and grain yield (*P < 0.05, **P < 0.01, ***P < 0.001). a = 0–15, b = 15–30, c = 30–45, d = 45–60, e = 60–75, f = 75–90, g = 90–105 and h = 105–120 cm soil depth.

Under DS in 2010–11, the RLD of 15–30 and 30–45 cm soil depths had a positive direct contribution to grain yield compared to the two remaining soil depths at 35 DAS (Fig. 3A). This was close to the pattern seen in 2009–10 except for the difference in contributory depth. At 45 DAS, the direct effect of RLD of all the soil depths turned to become positive on grain yield except for the RLD at the ultimate soil depth, 60–75 cm. The RLD at 0–15 and 15–30 cm soil depths had also shown a significant positive correlation with grain yield at p = < 0.01 level. RLD measured at 55 DAS had followed the same pattern of contribution as that at 45 DAS except for the enhanced levels of contribution from such contributory soil layers. RLD at 0–15, 15–30, 30–45 and 45–60 cm soil depths had significant positive correlations with grain yield that ranged in significance level from p = < 0.001 to p = < 0.05. At 65 DAS, the RLD at 15–30, 45–60 and 60–75 had a direct positive contribution to grain yield and the contribution of RLDs from remaining four soil depths were found to be negative but largely small. At this stage, RLDs at 45–60 and 60–75 cm soil depths had a strong significant correlation with grain yield at p = < 0.001 level. Though the direct contribution of roots of 30–45 is less negative or null, a positive significant correlation had appeared. A similar type of indirect positive contribution can also be seen by the RLD of 75–90 cm in translating a null direct effect in to a positive correlation coefficient at p= < 0.01 level. At 75 DAS, the path coefficients of RLD from all the soil depths other than 15–30 and 105–120 cm had shown positive direct contribution to grain yield (Fig. 3A). The RLD of 45–60 cm soil depth had the highest direct positive contribution followed by RLD at 75–90 and 60–75 cm soil depths. At this growth stage, the RLD at 45–60, 60–75 and 75–90 cm soil depths showed a significant positive correlation with grain yield with significance levels ranging from p = < 0.01 to p = < 0.001. At 90 DAS, the RLD at 0–15, 45–60, 60–75 and 105–120 cm soil depths alone had a direct positive contribution on grain yield. At this stage, the RLD of 45–60 cm soil depth had the highest direct positive contribution to grain yield followed by RLD at 105–120, 0–15 and 60–75 cm soil depths. RLD at 105–120 cm soil depth alone had a significant positive correlation with the grain yield at p = < 0.05 level.

Under OI in 2010–11, the RLD of 15–30 cm soil depth had a positive contribution to grain yield and this has emerged into a significant and positive correlation with grain yield in spite of some large negative contribution from RLD at 0–15 cm soil depth at 35 DAS (Fig. 3B). Another interesting observation at this stage was the complete absence of roots in the 45–60 cm soil layer while under DS there were roots (data not shown). This crop received the first treatmental irrigation five days before and this irrigation clearly seemed to slow down the progression of RDp. At 45 DAS, the overall positive correlation coefficients seen across most of the depths under DS were not noticeable but the positive coefficients were limited to roots of 15–30 cm depth (Fig. 3B). The major contribution of the RLD from the 15–30 soil depth emphasizes the importance of this soil layer in grain yield determinacy. At 55 DAS, RLD at 30–45 cm soil depth had a high direct and significant contribution to the grain yield and this significant contribution pattern was also followed by the roots at soil depths 60–75 and 15–30 cm. The RLD at 45–60 cm soil depth has presented a clear case of negative direct contribution to grain yield but despite that the correlation of overall RLD with the grain yield became highly significant. At 65 DAS, a major direct and positive contribution had been noticeable by the RLD at 15–30, 30–45 and 75–90, cm soil depths to the grain yield and also the RLD from these depths were correlated with the grain yield at p levels ranging from <0.05 to <0.001 (Fig. 3B). In addition, RLD of soil depth 60–75 cm had a significant correlation with grain yield at p < 0.05 level. Also at this stage, the depth that contributed to a large negative direct path coefficient was limited to the 0–15 cm soil depth. At 75 DAS, RLD from all the depths except 60–75 and 105–120 cm soil depths had a positive direct contribution to grain yield. The RLD of 45–60 cm soil depth had the highest direct positive contribution to grain yield followed by 15–30, 75–90, 30–45 and 0–15 cm soil depths. At this growth stage, the RLD at 15–30, 45–60, 60–75, 75–90 and 105–120 cm soil depths showed a significant positive correlation with the grain yield ranging from p = < 0.05 to p = < 0.01. At 90 DAS, RLD of soil depths 15–30, 60–75 and 90–105 cm had exhibited a positive direct contribution to grain yield (Fig. 3B). RLD of 90–105 cm soil depth had the highest direct positive effect on grain yield followed by RLD of 60–75 and 15–30 cm soil depths. However, RLD at 60–75 cm soil depth alone had shown a significant positive correlation with the grain yield at p = < 0.001 level.

**Fig. 4:**
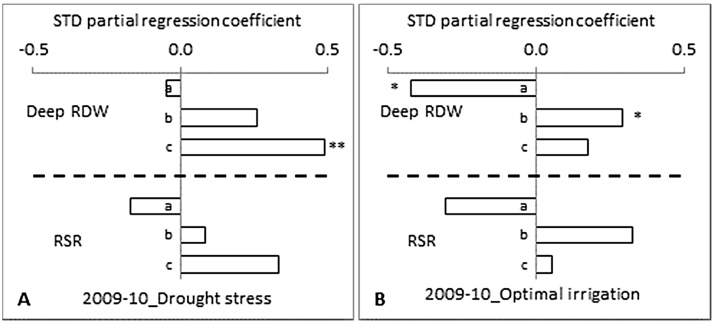
Standardized partial regression coefficients of deep root dry weight (Deep RDW) and root:shoot ratio (RSR) measured at different days after sowing (DAS) on grain yield at maturity of 12 diverse genotypes of chickpea both under (A) drought stress and (B) optimal irrigation in a Vertisol during 2009–10 post-rainy season. The different number of asterisks on top of the bars denotes the different level of significant correlation between RLD and grain yield (*P < 0.05, **P < 0.01). a = 35, b = 50 and c = 80 days after sowing.

**Fig. 5:**
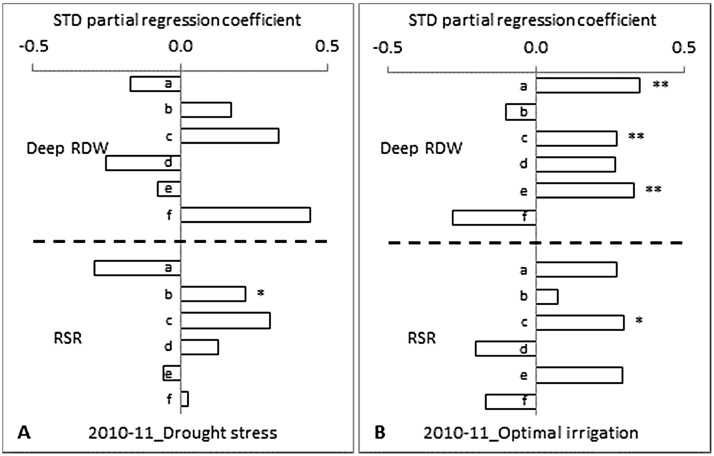
Standardized partial regression coefficients of deep root dry weight (Deep RDW) and root:shoot ratio (RSR) measured at different days after sowing (DAS) on grain yield at maturity of 12 diverse genotypes of chickpea both under (A) drought stress and (B) optimal irrigation in a Vertisol during 2010–11 post-rainy season. The different number of asterisks on top of the bars denotes the different level of significant correlation between RLD and grain yield (*P < 0.05, **P < 0.01). a = 35, b = 45, c = 55, d = 65, e = 75 and f = 90 days after sowing.

Under DS, the correlation coefficients of deep RDW and RSR were positive at all different sampling stages except at 35 DAS for deep RDW and 65 DAS for RSR in 2009–10 (Fig. 4A) and, at 35 DAS for deep RDW in 2010–11 (Fig. 5A). The deep RDW measured at 80 DAS had a highest positive direct effect on grain yield, with a significant positive correlation at p = < 0.01 level, followed by 50 DAS in 2009–10 and, it was highest at 90 DAS, with a significant positive correlation at p = < 0.05 level, followed by 55 and 45 DAS in 2010–11 (Fig. 4A and Fig. 5A). For RSR, it was the highest at 80 DAS followed by 50 DAS in 2009–10 and, it was 55 DAS followed by 45, 65 and 90 DAS in 2010–11. RSR measured at 45 DAS had a significant positive correlation with the grain yield at p = < 0.05 level.

Under OI, the correlation coefficient of deep RDW and RSR were positive at all the different sampling stages except at 35 DAS for deep RDW and 35 and 80 DAS for RSR in 2009–10 (Fig. 4B) and, at 90 DAS for RSR in 2010–11 (Fig. 5B). The deep RDW measured at 50 DAS had the highest positive direct effect on grain yield, with a significant positive correlation at p = < 0.05 level, followed by 80 DAS in 2009–10 and, it was the highest at 75 DAS followed by 55 and 65 DAS in 2010–11 (Fig. 4B and Fig. 5B). Deep RDW measured at 55 and 75 DAS had a significant positive correlation with grain yield at p = < 0.01 level. This significant correlation seems to express the peak (but temporary) stress occurrence as it was measured day or hours before the supplemental irrigation. The RSR measured at 50 DAS had the highest positive direct effect on grain yield followed by 80 DAS in 2009–10 and, it was the highest at 55 DAS, with a significant positive correlation at p = < 0.05 level, followed by 75, 35 and 45 in 2010–11.

### 3.5 Contribution of crop phenology, shoot biomass and harvest index to grain yield

The correlation of crop phenology (days to 50% flowering and the maturity) with grain yield was negative across drought treatments and years except for days to maturity under OI in 2009–10 (data not shown). There were trends of positive association of shoot biomass at maturity with grain yield irrespective of the drought treatment but it was highly significant only under OI in 2010–11. HI had been very closely associated with grain yield under both drought regimes and years and also the contributions were positive and large at all environments.

## 4 Discussion

In this study, the focus of drought tolerance is on the ability to sustain greater biomass production and grain yield under a seasonally increasing water deficit, rather than the physiological aptitude for plant survival under extreme drought shock (Serraj and Sinclair, 2002). However, the current level of knowledge do not permit complete reliance either on the traits of drought tolerance or the grain yield. Because the current level of knowledge on the physiological traits or combination of traits that explain the grain yield under water-limited environments is not adequately consistent and conclusive and the performance of grain yield is unstable under the influence of G × E interactions. This status, demands a parallel measurement of both the traits and the grain yield and verify the traits versus grain yield association. Therefore in this study drought tolerance has been primarily measured as grain yield under DS. Apart from grain yield, shoot biomass production under DS was also considered as an alternate drought tolerance measure depending on the contextual relevance (Pinheiro et al., 2005, Kobata et al., 1996, Krishnamurthy et al., 2010).

Physiological traits that might help in adaptation to water-limited environments are unlikely to be universal and some will be important in one environment while detrimental in another (Richards, 2006, Tardieu, 2012). In general, traits responsible for drought tolerance, and particularly drought avoidance, in any genotype are likely to be different from another as plants adapt to DS through different mechanisms and with the help of many different traits (Richards, 2006, Ludlow and Muchow, 1990, Saxena and Johansen, 1990, Johansen et al., 1997, Soltani et al., 2000, Tardieu, 2012). Among these traits, root traits (RDp, RLD, RDW, root surface area, average root diameter, root volume, root hair density) were found to be the major contributors to drought tolerance (avoidance) under rainfed condition (Ludlow and Muchow, 1990, Saxena et al., 1993, Krishnamurthy et al., 2003, Kashiwagi et al., 2006, Kashiwagi et al., 2015, Subbarao et al., 1995, Turner et al., 2001, Passioura, 2006, Zhu et al., 2010, Uga et al., 2013). Also, there were findings that the timings of root growth matters so as to rationalize the available water for a successful completion of the life cycle (Passioura, 1976, Zaman-Allah et al., 2011). Thus, it became necessary to measure all the plant traits that are organizationally immediately next in order to grain yield and integrated over the whole crop growing period. As related traits, the root growth across various stages of crop (35, 50 and 80 DAS in 2009–10, and 35, 45, 55, 65, 75 and 90 DAS in 2010–11), with shoot biomass and HI at maturity were monitored. The association of all these traits with the grain yield was expected to give an indication of various possible trait combinations and their significance in contribution to drought tolerance. However, the root traits that this study mainly focused was average RLD, total RDW, deep RDW (RDp) and RSR, that had been earlier known as major contributing traits compared to the other root parameters. Also some amount of information was generated on the RDp using deep soil RDW as the methodology employed was efficient enough only to detect differences in increments of 15 cm soil depth.

### 4.1 Root responses across years

The in-season rainfall and the cloudy days influenced the soil drying pattern and the root turnover across the two years. In 2009–10 the terminal drought development was comparatively early and the overall season VPD was relatively higher and this had ensured an early enhancement of the RLD as seen at the flowering stage (50 DAS). But a relative delay in terminal drought setting in 2010–11 also delayed the attainment of maximum RLD to about 15 days. Also this had considerably enhanced the deep soil root dry weight as early as 35 DAS. The RSR was relatively less under drier year. Also this rapid development of drought in 2009–10 influenced the appearance of maximum differences among genotypes in rooting response.

### 4.2 Relative performance of root traits among genotypes under drought stress

Root traits measured in this study have shown good range of variation among the genotypes as seen in previous studies under both field and lysimetric condition (Serraj et al., 2004, Kashiwagi et al., 2005, Lalitha et al., 2015). Moreover, these root traits had clearly differentiated the drought tolerant genotypes from the sensitive ones but only the timing of dominant root growth varied among the tolerant genotypes. Most of the tolerant chickpea genotypes had displayed root growth vigor and deeper soil root proliferation at early to mid-growth period for better adaptation to drought as in other crops (Otoole and Bland, 1987, Silim and Saxena, 1993, Saxena et al., 1995, Singh et al., 1995, Kell, 2011). To be precise, highest range of differentiation among genotypes appeared for RLD at 50–55 days after sowing (early pod fill stage), for deep root proliferation from 50 to 75 DAS (approach of maturity) and for the RSR at 35 DAS. Deep RDW had also been seen here to be a drought adaptive expression and there were no big genotypic differences at early stages (35 DAS) for this trait as the opportunity of stress had yet to appear. But by 45 DAS the genotypic expressions in deep RDW started to appear.

In previous works, the traits RDp, RLD and RDW have been identified to be relevant and primary for drought avoidance and listed to confer grain yield advantage in chickpea under terminal DS environments (Subbarao et al., 1995, Turner et al., 2001, Kashiwagi et al., 2005, Kashiwagi et al., 2006, Kumar et al., 2007). The emphasis placed upon RDp as an important trait for deeper soil water extraction to enhance reproductive growth and grain yield under DS (Saxena et al., 1993, Krishnamurthy et al., 2003, Zhang et al., 2004, Kashiwagi et al., 2005, Kirkegaard et al., 2007, Purushothaman et al., 2016a) had been confirmed to be appropriate with this work. The genotype ICC 4958 had a strong root system as early as 35 DAS, flowered and matured about 10 days earlier than mean flowering time. This early flowering provided two critical advantages; low level-stress facilitated longer reproductive duration and a better soil water-supported rapid rate in partitioning to grains (Krishnamurthy et al., 2013, Purushothaman et al., 2014). Both the fast declining available soil moisture and the approach of days of high temperature set a ceiling to the length of the growth duration in this tropical environment (Johansen et al., 1997). Early flowering ensured the possibility of an extended reproductive duration and also the seed filling was less restrained with enough soil water. Therefore, this genotype responded partly as a drought escape and partly as a drought tolerant; remained stable across years but could not use extended growing period for achieving the top yield slot when the environments were favorable. The other early and strong rooting kabuli genotype ICC 8261 was medium in duration but it was one of the latest to flower among the tested genotypes. But this late flowering did not reduce the reproductive growth duration and thus lead to exposure to an intense stress levels at the final phase of the growth. This genotype possessed early strong root growth as good as ICC 4958 and moderate root growth at later stages, produced highest shoot biomass but failed to transform it in to grain yield (particularly under 2010–11, when the terminal drought was relatively acute,) due to a poor HI. The drought adaptation of kabuli’s to constantly receding soil moisture environments were not the best as these are more adapted to relatively higher rain fall regions in early season as evidenced by the warmer late reproductive phase canopy temperatures and the possession of broader and greater number of xylem vessels (Purushothaman et al., 2014, Purushothaman and Krishnamurthy, 2014). Kabuli’s in general also require a longer and warmer reproductive duration to fulfill their longer seed filling requirements compared to *desis* and in the absence of such long periods the HI or partitioning to grains gets reduced seriously affecting the grain yield (Krishnamurthy et al., 2010).

Both the highly drought tolerant genotypes, ICC 867 and ICC 14778, had displayed similar performance for all measured root traits except for a minor variation in their phenology and root growth vigor. Though both the genotypes were medium in their flowering, ICC 14778 was the latest in maturity compared to other genotypes. These two genotypes were poor in root growth at 35 DAS but developed a stronger root system similar to that of ICC 4958 and ICC 8261, at 65 DAS. But ICC 867 was little early in root proliferation than ICC 14778 as the root growth started to be exponential at the approach of 50% flowering. Compared to ICC 4958 these highly drought tolerant genotypes had the additional benefit of utilizing a relatively extended growth period particularly in the second year, with an active root support at later stages, for achieving top yield under DS. These genotypes were able to utilize the whole season that the available soil water could permit with a conservative early root growth and a rapid later growth producing a matching shoot biomass and the best HI converting most of the shoot biomass into grain yield. One another tolerant genotype, ICC 3325, mimicked the performance of highly drought tolerant genotype ICC 14778, in root growth pattern but produced slightly less shoot biomass as in 2009–10 or HI as in 2010–11. The greater root proliferation and RDp at the vegetative stage seems to support an enhanced soil water uptake and vegetative biomass production leading to early ground cover. At the same time, maintaining similar level of root proliferation and RDp during the reproductive stage at deeper soil layers was found to benefit better partitioning rate through deep soil mining of water (Passioura, 1983, Passioura and Angus, 2010, Kell, 2011, Krishnamurthy et al., 2013, Cutforth et al., 2013, Wasson et al., 2014). The other tolerant genotype ICC 14799 had displayed above average root growth across both vegetative and reproductive stages of growth, maintained greater RSR, produced greater shoot biomass comparable to the highly drought tolerant genotypes but with a relatively moderate HI.

The small rooting genotypes, ICC 283 and ICC 1882, were early to medium in flowering and maturity and were the next early genotypes after ICC 4958 and Annigeri. These genotypes possessed small root system at 35 DAS and had a higher root growth at pod initiation stage in ICC 283 and at pod filling stage in ICC 1882. But both the genotypes had tended to produce higher root growth to achieve a moderate shoot biomass and grain yield with relatively higher HI. The highly drought sensitive genotypes ICC 3776 and ICC 7184 were medium in duration and were late among the genotypes tested. These had displayed poor root growth throughout their life cycle and one among them produced very poor shoot biomass in 2010–11 and had a very low HI. Among the studied genotypes, these two drought sensitive genotypes had produced the least shoot biomass seemingly due to limited soil water uptake (Purushothaman et al., 2016a) and restricted transpiration as a consequence of their poor root growth. The best adapted genotype Annigeri was the next earliest to flower and mature after ICC 4958 completing its life cycle at least 7 days before the other genotypes. This genotype possessed early moderate root growth both in terms of RDp and root proliferation, and moderate shoot biomass production and high grain yield through a maximum HI. The other best adapted genotype ICCV 10 was found to be the best yielder among the tested genotypes. This genotype had been characterized with a moderate root growth at the early stages, above-average root growth at reproductive stages (after 50 days of growth), moderate shoot biomass production and the highest HI making it to achieve the top grain yield slot. Therefore, better shoot biomass production and greater HI seem to be equally important for better drought tolerance and it is apparently achievable through a prolific and deep root system in chickpea and few other crops (Chapman et al., 1993, Blum, 2009, Chloupek et al., 2010, Kashiwagi et al., 2015). But this prolificacy need to be comprehended as a relative term and applicable only among the legumes because cereals are known to be equipped with 5 to 10 times more root length for uptake of about the same quantity of soil water (Hamblin and Tennant, 1987, Gregory and Eastham, 1996, Sandana and Pinochet, 2015).

### 4.3 Root traits contribution to drought tolerance

Rooting depth and proliferation depends on the gravitropism and hydrotropism as the root tip drives the gravitropic response in an auxin-dependent mechanism that alters cell elongation (Swarup and Bennett, 2009). There is a general agreement that root contribution to drought tolerance is through an effective utilization of soil moisture by the crop during its life cycle. Moreover, the crop water uptake in different soil layers was found to be different from one time to another along with the crop age. The amount of soil water depletion of a particular soil depth is maximized linearly when the amount of root proliferation increased in the same soil depth (Krishnamurthy et al., 1998, Sinclair et al., 2010, Purushothaman et al., 2016a). The pattern of soil water extraction is the continued progression from the surface layer to the deeper soil zones (Zhang et al., 2004, Purushothaman et al., 2016a). At the early vegetative stage (35 DAS), when plenty of stored soil water was still available even under DS, the path coefficients indicated that the roots (RLD) of soil depths 30–45 cm as the most active and decisive in causing useful differences in soil water uptake and drought tolerance (Purushothaman et al., 2016a). Whereas, under OI in 2010–11, when this treatment had already received the first irrigation, the water uptake at the 15–30 cm soil depth was critical and its association with grain yield was apparent. Similarly at the reproductive stage the most contributing soil layer seems to be 45–90 cm. The most successful genotypes such as ICC 867 and ICC 14778 mined this soil layer more than the other genotypes, particularly at 60–75 cm. Also the latest maturing genotype and medium duration genotype ICCV 10 used its root strength more at the 60–75 and 75–90 cm soil layers. Therefore, it is very clear that a competitive soil water use through root system capabilities tend to reflect in the drought tolerance supporting either the shoot biomass production or an effectively rapid rate of partitioning or both. However, the period of association between root and grain yield through soil water extraction might differ among genotypes and crops and it mainly depends on the genetic makeup and adaptation strategies of the genotypes and the soil moisture availability (Zhang et al., 2000).

In this study, the contribution of roots from 0 to 15 cm soil depth to grain yield at early vegetative stage was not consistent across years and the path coefficients were largely negative in both drought treatments and years. This inconsistency or null relation could have happened due to the variation in soil moisture loss through evaporation governed more by the vapor pressure deficit variations (French and Schultz, 1984, Siddique et al., 1990), as this layer comes in direct contact with the dry air/solar radiation. Chickpea plant has only a partial access to the soil water from this layer but a major quantity can be expected to be utilized in the very early growth stage (Kashiwagi et al., 2006, Kashiwagi et al., 2015). During the cropping season, it was estimated that about 28–35% of evapo-transpiration loss of chickpea and lentil is through direct soil evaporation (Zhang et al., 2000) and it is close to the value for other cereals and legumes (French and Schultz, 1984, Siddique et al., 1990, Loss et al., 1997). The negative path coefficients of the roots of 0–15 cm soil depth with the grain yield are most likely due to the early use of soil water and the subsequent early death and loss of secondary roots. Such developments preceded the first sample time, 35 DAS. Enormous root turnover had been observed in rain-fed chickpea particularly at the surface soil layers in response to the receding soil water (Krishnamurthy et al., 1996).

Genotypes that invest in higher RLD to utilize maximum soil water effectively competing with evaporation at this stage had been found to be critical for early shoot growth vigor via increased transpiration (Sponchiado et al., 1989, White and Castillo, 1989, Kashiwagi et al., 2006, Kashiwagi et al., 2015). This early growth vigor improved the ground cover and could reduce soil water evaporation to a significant level, ensuring better soil water availability for later use (Keatinge and Cooper, 1983, Morison et al., 2008). At 35 DAS, the genotype ICC 4958 produced consistently the maximum root proliferation at both surface (0–15 cm) and total soil depth (0–60 cm) across years and expected to leave a very minimal soil water at the surface for loss by evaporation. The best adapted genotype Annigeri also had a consistent behavior as seen in ICC 4958 at the soil depth of 0–15 cm. Root vigor and shoot vigor are mutually beneficial to each other like for the high shoot growth, crops rely on maximum soil water extraction through its vigorous and deep root system (Pinheiro et al., 2005, Kobata et al., 1996). Consequently, root vigor in turn relies on photo-assimilates to root tips for growth (Boyer et al., 2010), suggesting that manipulation of shoot growth would provide extra resources to root growth (Wasson et al., 2014). Genotypes vary in the timing of enhanced root growth expression as some of genotypes express early while some others express late. Though the timing of dominant root growth varies from one genotype to other, such greater root growths were associated with the grain yield advantages. Early maturing genotypes were greater in root vigour and it is favorable, in terms of crop productivity, in the environments where the growing season is short and terminal DS predominates. At the early stages of crop growth, the drought tolerant (ICC 3325, ICC 283, ICC 1882) and the highly drought tolerant genotypes (ICC 14778 and ICC 867) were comparatively poor in root expression and conservative in soil moisture uptake (Purushothaman et al., 2016a). Consequently, these genotypes did not utilize the available surface layer soil moisture fully. But these showed an urge to descent their distal roots towards the deeper soil layer with better root proliferation to extract the available soil moisture. Such increase in root growth at the deeper soil layer at later stages is critical to match the increasing transpiration demand in response to increasing atmospheric vapor pressure deficit under terminal DS (Blum, 2009, Lopes and Reynolds, 2010, Wasson et al., 2012). However, the mid-duration enhanced root growth can be more desirable than the late root growth as seen in ICC 14778, ICC 867, ICC 283, ICC 3325 and ICC 1882 as it cannot be beneficial, if the stored soil water is poor at the deeper soil layers or the soils are relatively coarse in texture or the soil is shallow. The genotypes that had produced larger root system, irrespective of their growth phase, also produced better shoot biomass compared to the drought sensitive genotypes ICC 7184 and ICC 3776 in this study. But the reproductive success depended (the biomass partitioning) more on the ability of the genotypes to match their phenology (by being plastic) to the soil water availability through an enhanced root activity.

Contribution of whole root system to grain yield at 35 DAS was shown to be high in chickpea (Kashiwagi et al., 2006) but the lack of consistency of such association across environments makes the breeders to consider root traits as hard to handle in the drought tolerance breeding programs (Vadez et al., 2008, Zaman-Allah et al., 2011). In this study, the measured root traits had been seen to offer a significant positive contribution to grain yield and this contributions were found to arise from particular rooting depths. Though, this depth level contribution of root traits to grain yield was highly consistent across stages and environments it was also found to move progressively to deeper soil depths with the increase in crop duration. Root growth pattern as the soil water recedes is a dynamic process that involves complex interactions among atmosphere, plant and soil (Landsberg and Fowkes, 1978, Draye et al., 2010). Therefore it often becomes difficult to trace a meaningful association of roots and grain yield.

Root distribution (in terms of RLD) differed among the soil depths and the genotypes across growth stages and it was found to be the highest in the surface soil layers (Zaman-Allah et al., 2011, Zarebanadkouki et al., 2013). At early stages of plant growth in this study, the genotypic differences were found to be high in surface soil layer root production (0–15 cm) and contribute to grain yield production apparently through better soil water extraction. But at the mid to late reproductive stages (65 DAS) of crop growth, roots from soil depth 0–15 cm started to show a negative contribution to grain yield likely due to two possibilities 1) the early root death in this layer immediately following soil drying and 2) relatively greater root distribution at this stage of late duration genotypes that suffered adverse effects on their reproductive success due to enhanced terminal DS and a consequent poor harvest index (Kashiwagi et al., 2015). This observation leads to a suggestion that, the existence of denser root in a particular soil depth may not contribute to greater drought yields unless it leads to greater soil water uptake and matching well with the growth and partitioning needs. Therefore, the contribution of roots present at 0–15 cm soil depth goes unnoticed. In such circumstance, roots from subsequent soil depths (30–90 cm) take up the major role in contribution to grain yield as seen in this study. The drying soil surface seems to reduce the size of shallow roots and enhance the deeper root production by redirecting the photo-assimilates to the primary roots which grew deeper in to the soil and result in increased RLD (Blum and Ritchie, 1984, Asseng et al., 1998, Wasson et al., 2014, Zarebanadkouki et al., 2013, Kashiwagi et al., 2015). Also there are genetic variations with clear timings on the occurrence of peak root growth. This was from the early stages in ICC 4958 and ICC 8261 but such a peak growth was after 65 DAS in the rest of the drought tolerant and the well adapted genotypes. Similar genetic variations of timing of root growth were shown in various other crops through a sustained transpiration and stomatal conductance measured by canopy temperature differences under DS (Blum et al., 1982, Kobata et al., 1996, Sanguineti et al., 1999, Araus et al., 2002, Pinheiro et al., 2005, Izanloo et al., 2008, Kashiwagi et al., 2008, Blum, 2009). In addition by direct measurements or through the estimates by modeling exercises in wheat or through empirical studies with various crops, the value and contribution of deep root to grain yield under DS in the field had been demonstrated well (Wasson et al., 2012).

At 80 and 75 DAS a massive significant contribution was provided by the roots inhabiting 75 to 105 cm soil layer in 2009–10 and 45 to 90 cm layer in 2010–11. Most of the drought tolerant genotypes had a strong root presence up to 105 cm soil depth, to have a complete access to available soil moisture at this stage. But such an access was achieved much earlier by the early maturing genotypes ICC 4958 and Annigeri. Faster, deeper and denser roots at the deeper soil layers with matching deeper soil moisture uptake generally coincide with grain development when crops are more vulnerable to DS (Passioura, 1983). Water use at this time has very high conversion efficiency in to grain as vegetative growth has ceased and all photo-synthates are diverted towards grain growth. Most of the increase in yield from late-season subsoil water use is through influencing the rate of partitioning (Passioura and Angus, 2010, Krishnamurthy et al., 2013). Contrastingly, the genotypes ICC 7184 and ICC 3776 had failed to have a complete access of soil moisture as these produced a very low root prolificacy even at this late stage explaining their drought sensitivity. The plants that have shallow root system are well demonstrated to have limited access to water uptake ensuring the lowest yield under rain-fed condition (Wasson et al., 2012).

Conservative use of water during the vegetative growth phase had been suggested to leave more water for the reproductive phase ensuring success in reproduction based on lysimetric observations (Zaman-Allah et al., 2011, Pantuwan et al., 2002). The rooting depth and the RLD were also not found to relate with the grain yield in these studies (Zaman-Allah et al., 2011). Four experimental conditions that were critically different between Zaman-Allah et al. (2011) and the current work might clarify more on the field performance of chickpea roots, soil water use and the ultimate contribution of roots. Zaman-Allah et al. (2011) worked with the mini-lysimeters with limited stored soil water; the root system was harvested six-weeks after the imposition of DS and used genotypes that widely vary in phenology. Also this study had shown that the grain yield to closely follow the water use between 48 and 61 days of age. A plant growth beginning with initial suboptimal water compared to that of field would certainly artificially emphasize more on the importance of water that remains at the reproductive phase (Tardieu, 2012) but in the field the DS development can be expected to be relatively slow with further opportunity to explore deeper soil zones. The root system is prone to sloughing in dry soils and in their work it was harvested six weeks after drought imposition and therefore the RLD is expected to reduce substantially with no relation to previous water uptake or biomass productivity. Use of widely varying growth duration genotypes would influence greater variation in root growth and active soil water use depending on the developmental stages and would shift the importance more on partitioning rather than shoot biomass productivity.

## 5 Conclusion

The present work has displayed a clear differentiation in temporal and spatial root growth among genotypes and these differences, both as deep root biomass and the root length density, had explained the drought response of chickpea. Roots were highly adaptive to drought. Roots were seen to be more prolific and to reach deeper soil zones responding to drought. Genotypes varied in their timing of active root growth. The drought tolerant and well-adapted genotypes had comparatively greater root growth than the drought sensitive throughout their life. The drought tolerant genotypes comprised of both drought escape types and the drought avoidant ones. The drought avoidant genotypes were strong in root growth either at the vegetative or the reproductive growth stages. But the highly drought tolerant genotypes such as ICC 14778 and ICCV 10 were clearly strong in late reproductive growth phase root growth and also had the best partitioning ability and the best grain yields under drought stress. Therefore the pattern of soil moisture depletion from the profile can heavily influence the response ranking of the genotypes. On the basis of the appearance of highest range of variation among genotypes and the closeness of their association with grain yield, drought tolerance can be estimated closely through RLD at 50–55 DAS (early pod fill), deep root proliferation between 50 and 75 DAS (pod fill to approach of maturity) and RSR at 35 DAS.

## Figures and Tables

**Table 1:** Genotype mean, trial mean and analysis of variance of average root length density of 12 diverse genotypes of chickpea at different days after sowing (DAS) both under drought stress and optimal irrigation in a Vertisol during 2009–10 and 2010–11 post-rainy seasons.

	Average root length density (cm cm^−3^)								
	2009–10			2010–11					
Genotypes/treatment	0–60_ 35DAS	0–90_ 50DAS	0–120_ 80DAS	0–60_ 35DAS	0–75_ 45DAS	0–90_ 55DAS	0–105_ 65DAS	0–120_ 75DAS	0–120_ 90DAS
Drought stress									
ICC 4958	0.248	0.428	0.249	0.213	0.319	0.323	0.343	0.355	0.164
ICC 8261	0.233	0.420	0.282	0.196	0.280	0.264	0.338	0.436	0.214
ICC 867	0.161	0.379	0.288	0.098	0.281	0.262	0.346	0.424	0.187
ICC 3325	0.206	0.332	0.286	0.140	0.253	0.279	0.411	0.471	0.262
ICC 14778	0.201	0.373	0.295	0.093	0.261	0.232	0.383	0.408	0.180
ICC 14799	0.217	0.394	0.289	0.176	0.242	0.342	0.392	0.435	0.221
ICC 1882	0.199	0.323	0.284	0.121	0.246	0.280	0.373	0.435	0.154
ICC 283	0.175	0.319	0.252	0.188	0.199	0.280	0.391	0.398	0.256
ICC 3776	0.157	0.326	0.265	0.088	0.198	0.188	0.300	0.330	0.138
ICC 7184	0.175	0.336	0.261	0.124	0.198	0.164	0.264	0.322	0.195
Annigeri	0.219	0.369	0.240	0.192	0.268	0.294	0.338	0.382	0.167
ICCV 10	0.191	0.412	0.284	0.149	0.264	0.276	0.346	0.437	0.200

Mean	0.199	0.368	0.273	0.148	0.251	0.265	0.352	0.403	0.195
S.Ed (±)	0.007	0.017	0.010	0.006	0.013	0.012	0.016	0.014	0.015
α-level	***	***	***	***	***	***	***	***	***
Heritability (h^2^_b_)	0.904	0.782	0.661	0.972	0.832	0.915	0.809	0.871	0.802

Optimal irrigation									
ICC 4958	0.300	0.419	0.252	0.164	0.293	0.284	0.462	0.273	0.273
ICC 8261	0.324	0.364	0.302	0.150	0.246	0.298	0.464	0.278	0.294
ICC 867	0.198	0.352	0.258	0.113	0.249	0.259	0.435	0.299	0.411
ICC 3325	0.249	0.326	0.226	0.120	0.265	0.283	0.495	0.332	0.409
ICC 14778	0.224	0.324	0.277	0.091	0.199	0.299	0.407	0.304	0.239
ICC 14799	0.244	0.363	0.255	0.116	0.242	0.298	0.415	0.345	0.373
ICC 1882	0.230	0.241	0.240	0.118	0.245	0.267	0.396	0.283	0.292
ICC 283	0.212	0.267	0.225	0.131	0.217	0.240	0.415	0.302	0.299
ICC 3776	0.195	0.281	0.218	0.111	0.181	0.172	0.360	0.268	0.351
ICC 7184	0.212	0.293	0.237	0.081	0.211	0.159	0.342	0.284	0.355
Annigeri	0.223	0.364	0.232	0.109	0.231	0.285	0.424	0.292	0.384
ICCV 10	0.204	0.364	0.279	0.134	0.224	0.293	0.445	0.335	0.306

Mean	0.235	0.330	0.250	0.120	0.233	0.261	0.422	0.300	0.332
S.Ed (±)	0.008	0.014	0.011	0.006	0.008	0.015	0.016	0.009	0.014
α-level	***	***	***	***	***	***	***	***	***
Heritability (h^2^_b_)	0.941	0.900	0.759	0.896	0.913	0.874	0.824	0.827	0.910

Genotype × Drought treatment									
α-level	*	*	*	*	*	ns	*	*	*

S.Ed (±) = Standard error of the difference.

**Table 2:** Trial mean and analysis of variance of deep root dry weight of 12 diverse genotypes of chickpea at different days after sowing (DAS) both under drought stress and optimal irrigation in a Vertisol during 2009–10 and 2010–11 post-rainy seasons.

Genotypes/treatment	Deep root dry weight − 2009–10			Deep root dry weight − 2010–11					
	35 DAS	50 DAS	80 DAS	35 DAS	45 DAS	55 DAS	65 DAS	75 DAS	90 DAS
Drought stress									
Mean	10.7	17.3	16.1	6.51	17.9	21.5	34.8	26.3	16.4
S.Ed (±)	1.86	4.55	3.23	1.31	4.05	6.80	7.47	4.76	6.44
α-level	***	**	***	***	***	***	***	***	***
Heritability (h^2^_b_)	0.715	0.510	0.658	0.553	0.593	0.556	0.598	0.850	0.534

Optimal irrigation									
Mean	12.6	8.90	5.58	4.41	6.82	5.73	15.7	12.7	18.3
S.Ed (±)	1.04	1.74	1.76	0.984	1.38	1.16	4.72	2.28	5.67
α-level	***	*	ns	***	***	***	**	***	**
Heritability (h^2^_b_)	0.926	0.387	0.183	0.679	0.717	0.680	0.475	0.568	0.430

S.Ed (±) = Standard error of the difference.

**Table 3:** Genotype mean, trial mean and analysis of variance of root:shoot ratio of 12 diverse genotypes of chickpea at different days after sowing (DAS) both under drought stress and optimal irrigation in a Vertisol during 2009–10 and 2010–11 post-rainy seasons.

Genotypes/treatment	Root:shoot ratio − 2009–10			Root:shoot ratio − 2010–11					
	35 DAS	50 DAS	80 DAS	35 DAS	45 DAS	55 DAS	65 DAS	75 DAS	90 DAS
Drought stress									
ICC 4958	0.382	0.200	0.055	0.918	0.666	0.327	0.277	0.189	0.065
ICC 8261	0.337	0.212	0.066	1.036	0.609	0.301	0.316	0.186	0.072
ICC 867	0.216	0.163	0.077	0.773	1.163	0.391	0.306	0.194	0.064
ICC 3325	0.287	0.163	0.062	1.367	0.796	0.383	0.400	0.250	0.120
ICC 14778	0.576	0.272	0.128	0.581	0.749	0.333	0.407	0.217	0.072
ICC 14799	0.619	0.296	0.110	1.743	0.847	0.475	0.468	0.245	0.102
ICC 1882	0.283	0.187	0.094	1.217	0.858	0.374	0.304	0.190	0.050
ICC 283	0.314	0.149	0.082	1.800	0.536	0.397	0.332	0.191	0.089
ICC 3776	0.149	0.132	0.069	0.528	0.480	0.275	0.262	0.186	0.051
ICC 7184	0.246	0.157	0.076	1.203	0.823	0.323	0.294	0.194	0.070
Annigeri	0.271	0.128	0.057	1.243	0.683	0.388	0.314	0.195	0.064
ICCV 10	0.237	0.220	0.096	1.015	0.814	0.337	0.280	0.187	0.067

Mean	0.326	0.190	0.081	1.119	0.752	0.359	0.330	0.202	0.074
S.Ed (±)	0.046	0.022	0.009	0.155	0.104	0.045	0.027	0.017	0.012
α-level	***	***	***	***	***	*	***	**	***
Heritability (h^2^_b_)	0.852	0.774	0.789	0.804	0.623	0.383	0.760	0.479	0.634

Optimal irrigation									
ICC 4958	0.496	0.319	0.066	0.748	0.582	0.289	0.293	0.145	0.081
ICC 8261	0.436	0.220	0.077	0.924	0.471	0.333	0.346	0.143	0.090
ICC 867	0.621	0.414	0.060	1.532	0.863	0.346	0.300	0.157	0.135
ICC 3325	0.370	0.300	0.071	1.202	0.765	0.322	0.370	0.168	0.111
ICC 14778	0.586	0.353	0.083	0.984	0.637	0.377	0.325	0.168	0.062
ICC 14799	0.464	0.255	0.072	1.097	0.784	0.395	0.340	0.199	0.119
ICC 1882	0.448	0.197	0.064	1.008	0.669	0.301	0.227	0.126	0.090
ICC 283	0.488	0.247	0.057	1.847	0.732	0.299	0.251	0.144	0.077
ICC 3776	0.222	0.131	0.040	1.767	0.562	0.193	0.252	0.125	0.090
ICC 7184	0.492	0.312	0.055	1.044	0.792	0.181	0.257	0.153	0.112
Annigeri	0.315	0.324	0.048	0.789	0.813	0.281	0.274	0.156	0.102
ICCV 10	0.198	0.247	0.054	2.824	0.903	0.322	0.338	0.149	0.080

Mean	0.428	0.277	0.062	1.314	0.714	0.303	0.298	0.153	0.096
S.Ed (±)	0.061	0.049	0.006	0.431	0.120	0.034	0.017	0.010	0.007
α-level	***	***	***	**	*	***	***	***	***
Heritability (h^2^_b_)	0.730	0.556	0.751	0.485	0.316	0.666	0.813	0.713	0.841

S.Ed (±) = Standard error of the difference.

**Table 4:** Genotype mean, trial mean and analysis of variance of crop phenology, shoot biomass, grain yield and harvest index of 12 diverse genotypes of chickpea both under drought stress and optimal irrigation in a Vertisol during 2009–10 and 2010–11 post-rainy seasons.

Genotypes/treatment	Days to 50% flowering		Days to maturity		Shoot biomass (kg ha^−1^)		Grain yield (kg ha^−1^)		Harvest index (%)	
	2009–10	2010–11	2009–10	2010–11	2009–10	2010–11	2009–10	2010–11	2009–10	2010–11
Drought stress										
ICC 4958	38	33	79	83	3507	3680	1915	1905	54.6	51.8
ICC 8261	48	52	97	95	4605	4133	1674	1131	36.3	27.3
ICC 867	48	47	90	90	3858	3871	2078	1878	54.9	48.6
ICC 3325	48	49	93	92	3480	3907	1752	1894	50.4	48.5
ICC 14778	52	52	96	93	4232	3822	2016	1911	48.2	50.0
ICC 14799	50	51	94	92	3844	3639	1734	1694	45.0	46.5
ICC 1882	45	43	89	93	3506	3636	1871	1797	53.6	49.4
ICC 283	45	41	87	86	3395	3198	1789	1535	52.7	48.0
ICC 3776	49	47	98	94	4091	3698	1628	1355	39.9	36.5
ICC 7184	50	44	100	91	3756	3339	1093	1078	29.1	32.3
Annigeri	41	35	82	87	3567	3554	1923	1873	53.9	52.7
ICCV 10	47	44	93	90	3669	3921	2069	2118	56.4	54.0

Mean	47	44.8	92	90.5	3792.5	3699.8	1795.2	1680.7	47.9	45.5
S.Ed (±)	0.800	0.480	2.20	0.820	285.0	134.3	102.4	71.1	2.29	1.21
α-level	***	***	***	***	**	***	***	***	***	***
Heritability (h^2^_b_)	0.945	0.991	0.839	0.922	0.425	0.681	0.807	0.935	0.902	0.971

Optimal irrigation										
ICC 4958	49	47	111	103	7116	6582	1894	3141	26.7	47.8
ICC 8261	53	55	115	107	7529	6740	1308	2183	17.4	32.5
ICC 867	51	51	111	103	7348	7215	2158	3205	29.2	44.5
ICC 3325	51	53	113	104	6846	7277	2086	3174	30.8	43.6
ICC 14778	54	54	112	103	6404	6345	2035	3134	32.2	49.4
ICC 14799	53	54	113	105	7378	7928	1842	3161	25.0	39.9
ICC 1882	51	49	114	95	6578	6918	1949	3194	29.8	46.3
ICC 283	51	49	113	104	6935	6436	1982	3094	28.9	48.4
ICC 3776	53	53	110	106	7653	7205	1529	2485	20.0	34.5
ICC 7184	53	53	112	106	6171	5652	1309	1876	21.2	33.2
Annigeri	50	50	114	103	7233	7280	1993	3597	27.6	49.6
ICCV 10	50	50	115	103	7682	7527	2362	4202	30.7	55.8

Mean	51.7	51.4	112.7	103.5	7072.7	6925.6	1870.5	3037.2	26.6	43.8
S.Ed (±)	1.04	0.540	0.930	1.92	369.0	381.3	149.6	89.87	2.12	1.89
α-level	**	***	***	***	*	***	***	***	***	***
Heritability (h^2^_b_)	0.519	0.931	0.624	0.567	0.463	0.581	0.741	0.969	0.752	0.907

Genotype × Drought treatment										
α-level	*	*	*	*	ns	ns	ns	*	ns	ns

S.Ed (±) = Standard error of the difference.
