# Supplementary material for: Root traits confer grain yield advantages under terminal drought in chickpea (Cicer arietinum L.)
Source: Field Crops Res. 2017 Feb 1;201:146–61. doi: 10.1016/j.fcr.2016.11.004 (PMC5221670; doi:10.1016/j.fcr.2016.11.004)
Supplement: Supplementary file 1 [file mmc1.docx]

**Supplementary Table 1**

The root, drought and canopy temperature reactions of the diverse genotypes and the checks (best adapted varieties) used in this study.

Germplasm Root strength Drought Canopy

S. No accession at 35 days age reaction ^(4)^ temperature ^(3)^

1 ICC 4958 Large ^(2)^ Moderately tolerant Cool

2 ICC 8261 Large ^(2)^ Moderately tolerant

3 ICC 867 Highly tolerant Cool

4 ICC 3325 Tolerant Cool

5 ICC 14778 Highly tolerant Cool

6 ICC 14799 Tolerant Cool

7 ICC 1882 Small ^(2)^ Tolerant

8 ICC 283 Small ^(2)^ Tolerant

9 ICC 3776 Highly sensitive Warm

10 ICC 7184 Highly sensitive Warm

11 Annigeri Tolerant, adapted variety

12 ICCV 10 Large ^(1)^ Wider adapted variety

^(1)^ Ali et al., 2002; ^(2)^ Kashiwagi et al., 2005; ^(3)^ Kashiwagi et al., 2008; ^(4)^ Krishnamurthy et al., 2010.

**Supplementary Figure S1:** Weather during the crop growing seasons (November to March) of 2009-10 and 2010-11

**Supplementary Figure S2:** Changes in available soil moisture up to a soil depth of 1.2 m across the crop growing seasons of 2009-10 and 2010-11. Vertical bars denote standard error of differences (±)
